# Simulating Anisotropic quantum Rabi model via frequency modulation

**DOI:** 10.1038/s41598-019-40899-7

**Published:** 2019-03-14

**Authors:** Gangcheng Wang, Ruoqi Xiao, H. Z. Shen, Chunfang Sun, Kang Xue

**Affiliations:** Center for Quantum Sciences and School of Physics, Northeast Normal University, Changchun, 130024 China

## Abstract

Anisotropic quantum Rabi model is a generalization of quantum Rabi model, which allows its rotating and counter-rotating terms to have two different coupling constants. It provides us with a fundamental model to understand various physical features concerning quantum optics, solid-state physics, and mesoscopic physics. In this paper, we propose an experimental feasible scheme to implement anisotropic quantum Rabi model in a circuit quantum electrodynamics system via periodic frequency modulation. An effective Hamiltonian describing the tunable anisotropic quantum Rabi model can be derived from a qubit-resonator coupling system modulated by two periodic driving fields. All effective parameters of the simulated system can be adjusted by tuning the initial phases, the frequencies and the amplitudes of the driving fields. We show that the periodic driving is able to drive a coupled system in dispersive regime to ultrastrong coupling regime, and even deep-strong coupling regime. The derived effective Hamiltonian allows us to obtain pure rotating term and counter-rotating term. Numerical simulation shows that such effective Hamiltonian is valid in ultrastrong coupling regime, and stronger coupling regime. Moreover, our scheme can be generalized to the multi-qubit case. We also give some applications of the simulated system to the Schrödinger cat states and quantum gate generalization. The presented proposal will pave a way to further study the stronger anisotropic Rabi model whose coupling strength is far away from ultrastrong coupling and deep-strong coupling regimes in quantum optics.

## Introduction

The quantum Rabi model (QRM)^1–3^ is a fundamental model to describe the light-matter interaction, which has been at the heart of important discoveries of fundamental effects of quantum optics. When the ratio of coupling strength and mode frequency is much smaller than 1, rotating wave approximation (RWA) is valid and the QRM in this regime can be reduced to the Jaynes-Cummings (JC) model^4,5^, which has been used to describe the basic interactions in various systems^6–14^. Of particular interest is to implement the QRM in ultra-strong coupling (USC) regime (the coupling strength is comparable to the cavity frequency)^15–29^, and even deep-strong coupling (DSC) regime (the coupling strength exceeds the cavity frequency)^30^, in which RWA is not suitable and the counter-rotating term (CRT) cannot be neglected. This is because various effects induced by CRT appear in these regimes^31–42^. Such tremendous advances in experiments have also motivated various potential applications to quantum information technologies^43–47^. Although great progresses have been achieved, it is also very challenging to implement such model in USC and DSC regimes experimentally. The quantum simulation proposal provides us with an experimental accessible approach to implement the QRM in USC and DSC regimes, respectively^48–59^.

Recently, a generalized QRM with distinct RT and CRT coupling constants, which has been referred to anisotropic quantum Rabi model (AQRM), is attracting interests^60–66^. Due to such interesting characteristics, the AQRM has been utilized to study various theoretical issues, e.g., quantum phase transitions^67,68^, quantum state engineering^69^, quantum fisher information^70^, and so on. To date, people have proposed several methods to realize AQRM, which include the natural implementations of AQRM in quantum optics in a cross-electric and magnetic field^64^, electrons in semiconductors with spin-orbit coupling^70,71^, and superconducting circuits systems^72,73^. Meanwhile, quantum simulation methods with superconducting circuits^74^ and trapped ions^75^ have also been proposed. The AQRM provides us with a paradigm to understand the light-matter interaction and solid-state system. However, these implementations of AQRM are limited on the tunabilities, which motivate us to develop a frequency modulated method to realize a tunable AQRM in USC or even DSC regimes.

In this paper, we propose an effective method to simulate a tunable AQRM with a qubit coupled to a resonator in dispersive regime, and the transition frequency of the qubit is modulated by two periodic driving fields. The periodic driving have been widely used to modulate quantum systems^76–85^. We show that all the parameters in the effective Hamiltonian depend on the external driving fields. The frequencies of qubit and resonator for the simulated system can be adjusted by controlling the frequencies of the driving fields, while the anisotropic coupling coefficients of the RT and CRT are decided by the amplitudes of the driving fields. Our proposal to implement the AQRM has three features: (i) The effective Hamiltonian is controllable, and all the parameters can be tuned by controlling the external driving fields. (ii) We can drive the system from weak-coupling regime to USC regime and even DSC regime by tuning the frequencies and amplitudes of the driving fields. (iii) The ratio of coupling constants of RT and CRT can be controlled in a wide range of parameter space, which makes it possible to study the transitions from JC regime to anti-JC regime.

## The Derivation of the Effective Hamiltonian

**Figure 1:**
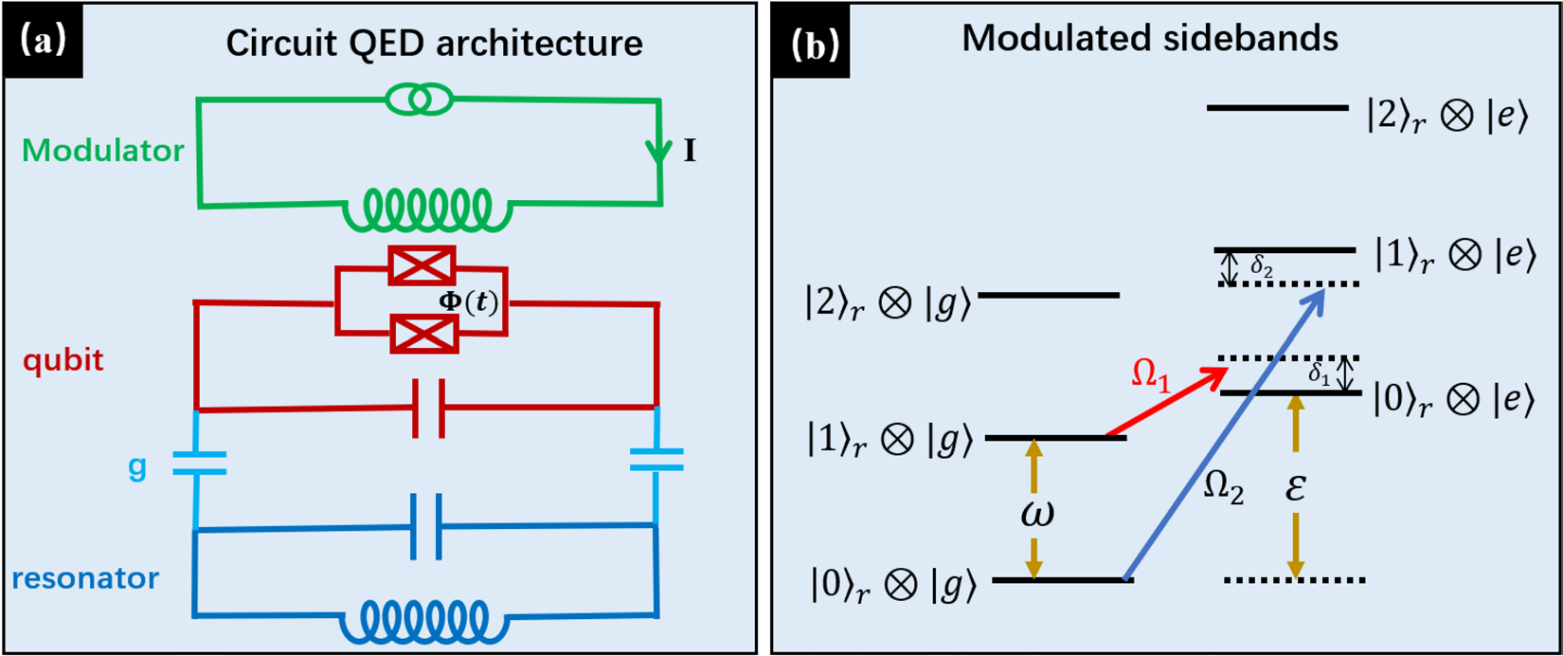
(**a**) The circuit QED architecture of the system: A transmon qubit is capacitively coupled to a LC resonator with frequency *ω*^99^. The transmon qubit, which is implemented with split junctions, can be modulated by the time-dependent flux generated by modulation circuit. The modulation Hamiltonian is shown in Eq. (2c). (**b**) The energy level of the modulated qubit-resonator system. Red and blue sidebands detunings of driving fields allow us to tune coupling constants of RT and CRT.

In this section, we consider a qubit coupled to a harmonic oscillator in dispersive regime, and the qubit is modulated by the periodic driving fields. Such setup can be realized in a variety of different physical contexts, such as trapped ions^6–9^, circuit QED^10–12^, cavity QED^13,14^, and so on. Here, we adopt a circuit QED setup to illustrate our proposal (the architecture is depicted in Fig. 1(a)). We consider a tunable transmon qubit, which is comprised of split junctions, is capacitively coupled to a LC resonator. Such split structure allows the qubit to be modulated by the magnetic flux through the pair junctions. The system is described by a time-dependent Hamiltonian as follows (we set *ħ* = 1)

1 $$\hat{H}(t)={\hat{H}}_{0}+{\hat{H}}_{{\rm{int}}}+{\hat{H}}_{d}(t),$$

where ${\hat{H}}_{0}$, ${\hat{H}}_{{\rm{int}}}$ and ${\hat{H}}_{d}(t)$ are given as follows

2a $${\hat{H}}_{0}=\omega {\hat{a}}^{\dagger }\hat{a}+\frac{\varepsilon }{2}{\hat{\sigma }}_{z},$$

2b $${\hat{H}}_{{\rm{int}}}=g(\hat{a}+{\hat{a}}^{\dagger }){\hat{\sigma }}_{x},$$

2c $${\hat{H}}_{d}=\sum _{j=1}^{{n}_{d}}\,{{\rm{\Omega }}}_{j}{\eta }_{j}\,\cos \,({{\rm{\Omega }}}_{j}t+{\varphi }_{j}){\hat{\sigma }}_{z},$$

where *ε* is the transition frequency of the tranmon qubit. ${\hat{\sigma }}_{\alpha }$ is the *α*-component of the Pauli matrices. *ω* is the frequency of the LC resonator. $\hat{a}$ $({\hat{a}}^{\dagger })$ is the annihilation (creation) operator. *g* is the coupling constant between the qubit and the bosonic field, and ${\hat{H}}_{d}(t)$ describes *n*_*d*_ periodic driving fields with frequencies Ω_*j*_ and normalized amplitudes *η*_*i*_. In this work, we consider *n*_*d*_ = 2 and the qubit coupled to the resonator in dispersive regime (*i.e*., $|g|\ll |{{\rm{\Delta }}}_{\pm }|$ with Δ_±_ = *ω* ± *ε*). Without periodic driving, the RT and CRT terms can be ignored in dispersive regime. This is because all terms are fast oscillating terms in the rotating framework. If we choose proper modulation frequencies and amplitudes such that the near resonant physical transitions are remained and far off resonant transitions can be discarded. Moving to the rotating frame defined by the following unitary operator

3 $${U}_{1}(t)=\exp (-i{\hat{H}}_{0}t-i\sum _{j=1}^{2}\,{\eta }_{j}\,\sin \,({{\rm{\Omega }}}_{j}\,t+{\varphi }_{j}){\hat{\sigma }}_{z}),$$

we obtain the transformed Hamiltonian

4 $$\begin{array}{rcl}\hat{H}^{\prime} (t) & = & {U}_{1}^{\dagger }(t)\hat{H}(t){U}_{1}(t)-i{U}_{1}^{\dagger }(t)({\partial }_{t}{U}_{1}(t))\\  & = & g\hat{a}[{\hat{\sigma }}_{-}{e}^{-i{{\rm{\Delta }}}_{+}t}\,\exp (-i\sum _{j=1}^{2}\,{\eta }_{j}\,\sin \,({{\rm{\Omega }}}_{j}t+{\varphi }_{j}))\\  &  & +\,{\hat{\sigma }}_{+}{e}^{-i{{\rm{\Delta }}}_{-}t}\,\exp (i\,\sum _{j=1}^{2}\,{\eta }_{j}\,\sin \,({{\rm{\Omega }}}_{j}t+{\varphi }_{j}))]+{\rm{H}}.\,{\rm{c}}.\,,\end{array}$$

where ${\hat{\sigma }}_{\pm }=({\hat{\sigma }}_{x}\pm i{\hat{\sigma }}_{y})/2$. Using the following Jacobi-Anger expansion^86,87^

5 $$\exp \,(2i{\eta }_{j}\,\sin \,({{\rm{\Omega }}}_{j}t+{\varphi }_{j}))=\sum _{n=-\infty }^{+\infty }\,{J}_{n}(2{\eta }_{j})\,\exp \,[in({{\rm{\Omega }}}_{j}t+{\varphi }_{j})],$$

with *J*_*n*_(*x*) being the *n*-th order Bessel function of the first kind, we obtain

6 $$\hat{H}^{\prime} (t)=g[\alpha (t)\hat{a}{\hat{\sigma }}_{+}+\beta (t)\hat{a}{\hat{\sigma }}_{-}]+{\rm{H}}.\,{\rm{c}}.\,,$$

where

7 $$\begin{array}{rcl}\alpha (t) & = & \sum _{{n}_{1},{n}_{2}=-\infty }^{+\infty }\,{J}_{{n}_{1}}(2{\eta }_{1}){J}_{{n}_{2}}(2{\eta }_{2}){e}^{i({n}_{1}{\varphi }_{1}+{n}_{2}{\varphi }_{2})}{e}^{i{{\rm{\Omega }}}_{-}({n}_{1},{n}_{2})t},\\ \beta (t) & = & \sum _{{n}_{1},{n}_{2}=-\infty }^{+\infty }\,{J}_{{n}_{1}}(2{\eta }_{1}){J}_{{n}_{2}}(2{\eta }_{2}){e}^{-i({n}_{1}{\varphi }_{1}+{n}_{2}{\varphi }_{2})}{e}^{-i{{\rm{\Omega }}}_{+}({n}_{1},{n}_{2})t}.\end{array}$$

Here, Ω_±_(*n*_1_, *n*_2_) = Δ_±_ + *n*_1_Ω_1_ + *n*_2_Ω_2_. According to the RWA, only slowly varying terms appearing in *α*(*t*) and *β*(*t*) will dominate the dynamics. We should choose the suitable driving frequencies to obtain the rotating and counter-rotating interaction terms. We assume there is a small detuning *δ*_1_ (*δ*_2_) between Ω_1_ (Ω_2_) and the red (blue) sideband, and the definition of the detunings read

8 $${\delta }_{1}={{\rm{\Omega }}}_{1}-{{\rm{\Delta }}}_{-},\,{\delta }_{2}={{\rm{\Delta }}}_{+}-{{\rm{\Omega }}}_{2}.$$

The energy levels of the modulated system are shown in Fig. 1(b). Considering small detunings (*i.e*. $|{\delta }_{i}|\ll |{{\rm{\Delta }}}_{\pm }|$) and dispersive coupling regime (*i.e*. $|g|\ll |{{\rm{\Delta }}}_{\pm }|$), one can check that the RT and the CRT will contribute to the dynamics only for lowest oscillating frequencies Ω_−_(−1, 0) = −*δ*_1_ and Ω_+_(0, −1) = *δ*_2_, respectively. When the oscillating frequencies are much larger than the effect couplings, i.e., $|{{\rm{\Omega }}}_{+}({m}_{1},{m}_{2})|\gg |g{J}_{{m}_{1}}(2{\eta }_{1}){J}_{{m}_{2}}(2{\eta }_{2})|$ with (*m*_1_, *m*_2_) ≠ (0, −1) and $|{{\rm{\Omega }}}_{-}({q}_{1},{q}_{2})|\gg |g{J}_{{q}_{1}}(2{\eta }_{1}){J}_{{q}_{2}}(2{\eta }_{2})|$ with (*q*_1_, *q*_2_) ≠ (−1, 0), one may safely neglect these fast oscillating terms in Eq. (6). Then the dominant terms in Eq. (7) are $\alpha (t)\approx -\,{J}_{1}(2{\eta }_{1}){J}_{0}(2{\eta }_{2}){e}^{-i{\varphi }_{1}}{e}^{-i{\delta }_{1}t}$ and $\beta (t)\approx -\,{J}_{0}(2{\eta }_{1}){J}_{1}(2{\eta }_{2}){e}^{i{\varphi }_{2}}{e}^{-i{\delta }_{2}t}$, where we have used the relation *J*_−*n*_(*x*) = (−1)^*n*^*J*_*n*_(*x*) for integer *n*. Then these approximations lead to the following near resonant time-dependent Hamiltonian

9 $$\begin{array}{rcl}\hat{H}^{\prime} (t) & \approx  & ({\tilde{g}}_{r}\,\hat{a}{\hat{\sigma }}^{+}{e}^{-i({\delta }_{1}t+{\varphi }_{1})}+{\tilde{g}}_{cr}\,{\hat{a}}^{\dagger }{\hat{\sigma }}^{+}{e}^{i({\delta }_{2}t-{\varphi }_{2})})+{\rm{H}}.\,{\rm{c}}.\,,\end{array}$$

where the effective coupling strengths of RT and CRT are

10 $${\tilde{g}}_{r}=-\,g{J}_{1}(2{\eta }_{1}){J}_{0}(2{\eta }_{2}),\,{\tilde{g}}_{cr}=-\,g{J}_{0}(2{\eta }_{1}){J}_{1}(2{\eta }_{2}).$$

The Hamiltonian in Eq. (9) is the so-called AQRM in interaction picture with effective resonator frequency $\tilde{\omega }=({\delta }_{1}+{\delta }_{2})\mathrm{/2}$ and qubit transition frequency $\tilde{\varepsilon }=({\delta }_{2}-{\delta }_{1})\mathrm{/2}$. Defining the new rotating framework associated with the time-dependent unitary operator

11 $${U}_{2}(t)=\exp \,(i\tilde{\omega }{\hat{a}}^{\dagger }\hat{a}t+i\frac{\tilde{\varepsilon }}{2}{\hat{\sigma }}_{z}t),$$

we obtain the effective Hamiltonian with anisotropic coupling strengths for RT and CRT

12 $${\hat{H}}_{{\rm{eff}}}=\tilde{\omega }{\hat{a}}^{\dagger }\hat{a}+\frac{\tilde{\varepsilon }}{2}{\hat{\sigma }}_{z}+{\tilde{g}}_{r}(\hat{a}{\hat{\sigma }}_{+}+{\hat{a}}^{\dagger }{\hat{\sigma }}_{-})+{\tilde{g}}_{cr}(\hat{a}{\hat{\sigma }}_{-}{e}^{i\theta }+{\hat{a}}^{\dagger }{\hat{\sigma }}_{+}{e}^{-i\theta }),$$

where we have set *φ*_1_ = 0 and *φ*_2_ = *θ*. The anisotropic parameter *λ* is the ratio of RT and CRT coupling strengths (*i.e*., $\lambda ={\tilde{g}}_{cr}/{\tilde{g}}_{r}$). Thus we obtain a controllable AQRM. Below we analyze the parameters in our scheme. In our circuit QED setup, we consider the following realistic parameters^88,89^: the transition frequency of the transmon qubit is *ε* = 2*π* × 5.4 GHZ with the decay rate *κ* = 2*π* × 0.05 MHz, the resonator frequency is *ω* = 2*π* × 2.2 GHz with the loss rate *γ* = 2*π* × 0.012 MHz, and the coupling strength of the resonator and qubit is *g* = 2*π* × 70 MHz. We can check that the dispersive condition (i.e., $|g|\ll |{{\rm{\Delta }}}_{\pm }|$) is fulfilled. The frequency modulation can be implemented by applying proper biasing magnetic fluxes. The modulation parameters Ω_*i*_, *η*_*i*_ and *φ*_*i*_ can be chosen on demand by tuning the modulation fields. In circuit QED setups, the modulation frequency and modulation amplitude range from hundreds of megahertz to several gigahertz. It is reasonable to set the modulation amplitude *η*_*i*_Ω_*i*_ ranges from 0 to 2*π* × 10 GHz^16^. The detunings *δ*_*i*_ can be tuned from 0 to hundreds of megahertz to fulfill the condition $|{\delta }_{i}|\ll |{{\rm{\Delta }}}_{\pm }|$.

## The Simulation of QRM and AQRM in USC and DSC Regimes

To assess the robustness of our proposal in circuit QED system, we should consider the dissipation effects in the following discussions^88^. Considering the zero-temperature Markovian environments and large driven frequencies Ω_*j*_, the master equation governing the evolution of the system can be derived as follows^52^

13 $$\dot{\rho }=-\,i[\hat{H}(t),\rho ]+{ {\mathcal L} }_{q}[\rho ]+{ {\mathcal L} }_{r}[\rho ],$$

where ${ {\mathcal L} }_{q}[\rho ]=\frac{\kappa }{2}(2{\hat{\sigma }}_{-}\rho {\hat{\sigma }}_{+}-\rho {\hat{\sigma }}_{+}{\hat{\sigma }}_{-}-{\hat{\sigma }}_{+}{\hat{\sigma }}_{-}\rho )$ and ${ {\mathcal L} }_{r}=\frac{\gamma }{2}(2\hat{a}\rho {\hat{a}}^{\dagger }-\rho {\hat{a}}^{\dagger }\hat{a}-{\hat{a}}^{\dagger }\hat{a}\rho )$ are the standard Lindblad super-operators describing the losses of the system. To obtain the master equation in the framework of effective Hamiltonian, we set *U*(*t*) = *U*_2_(*t*)*U*_1_(*t*). Let $\tilde{\rho }(t)$ be the density matrix in the same framework with effective Hamiltonian. Inserting $\rho (t)=U(t)\tilde{\rho }(t){U}^{\dagger }(t)$ to the master Eq. (13), we obtain the following master equation

14 $$\dot{\tilde{\rho }}=-\,i[\hat{\tilde{H}}(t),\tilde{\rho }]+{ {\mathcal L} }_{q}[\tilde{\rho }]+{ {\mathcal L} }_{r}[\tilde{\rho }],$$

where $\hat{\tilde{H}}(t)={U}^{\dagger }(t)\hat{H}(t)U(t)-i{U}^{\dagger }(t)({\partial }_{t}U(t))$ is the total system Hamiltonian in the new rotating framework. We show that the Hamiltonian ${\hat{H}}_{{\rm{eff}}}$ in Eq. (12) is the approximation of $\hat{\tilde{H}}(t)$ under RWA. Here we consider the initial phase difference of the driving fields is *θ* = 0. The parameters *η*_1,2_ and the detuning of first sideband *δ*_1,2_ are tunable parameters. Such tunable parameters determine the parameters in the simulated system in Eq. (12). To verify the validity of the effective Hamiltonian in Eq. (12), we should study the fidelity of the evolution state. Let $|\tilde{\psi }(0)\rangle$ be an initial state in the new framework and the corresponding initial density matrix is $\tilde{\rho }(0)=|\tilde{\psi }(0)\rangle \langle \tilde{\psi }(0)|$. Substituting $\tilde{\rho }(0)$ into Eq. (12), we obtain the evolution density matrix $\tilde{\rho }(t)$. The ideal case can be obtained by solving the Schördinger equation governed by the effective Hamiltonian (12). We denote the ideal evolution state governed by the effective Hamiltonian (12) with $|\tilde{\psi }(t)\rangle$. Then the fidelity of the evolution state reads $F(t)=|\langle \tilde{\psi }(t)|\tilde{\rho }(t)|\tilde{\psi }(t)\rangle |$.

### The simulation of QRM

In this subsection, we will show the performance of the simulated QRM. To obtain equal effective RT and CRT coupling strengths (i.e., *λ* = 1), we need to adjust the normalized amplitude *η*_*i*_. A simple case is *η*_1_ = *η*_2_ = *η*. Then the simulated coupling strength ${\tilde{g}}_{r}={\tilde{g}}_{cr}$ and we denote the simulated coupling strength with $\tilde{g}=-\,g{J}_{0}(2\eta ){J}_{1}(2\eta )$. Assuming *θ* = 0, we can obtain the following tunable QRM

15 $${H}_{{\rm{QRM}}}=\tilde{\omega }{\hat{a}}^{\dagger }\hat{a}+\frac{\tilde{\varepsilon }}{2}{\hat{\sigma }}_{z}+\tilde{g}({\hat{a}}^{\dagger }+\hat{a}){\hat{\sigma }}_{x}.$$

The effective frequencies of resonator and qubit are determined by the detunings *δ*_*i*_. One can tuning the ratios of modulation amplitudes and frequencies to obtain different relative coupling strength.

**Figure 2:**
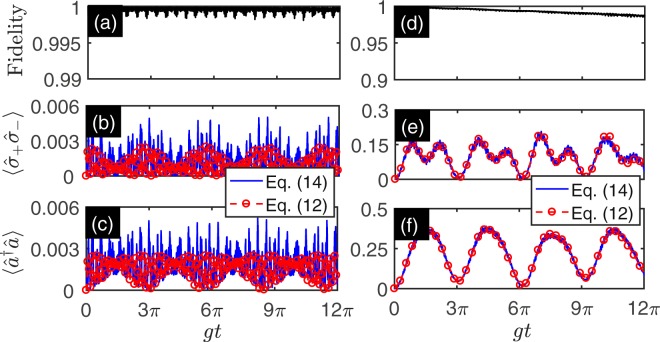
The fidelity and dynamics of the simulated QRM with effective coupling ratio $|\tilde{g}/\tilde{\omega }|=0.05$ (**a**–**c**) and $|\tilde{g}/\tilde{\omega }|=0.5$ (**d**–f) as a functions of evolution time. (**a**,**d**) Show the fidelity as a function of evolution time governed by the master equation in Eq. (14) and the simulated Hamiltonian given in Eq. (12). (**b**,**e**) Show the qubit excitation number $\langle {\hat{\sigma }}_{+}{\hat{\sigma }}_{-}\rangle$ as a function of evolution time. (**c**,**f**) Show the excitation number of the resonator $\langle {\hat{a}}^{\dagger }\hat{a}\rangle$ as a function of evolution time. The dynamics is governed by the master equation in Eq. (14) (solid blue line) and the simulated Hamiltonian given in Eq. (12) (red dashed line with circles). The red sideband modulation parameters are chosen as Ω_1_ = 2*π* × 3.2 GHz, and *η*_1_Ω_1_ = 2*π* × 2.296 GHz. The blue sideband modulation parameters are chosen as follows: Ω_2_ = 2*π* × 6.759 GHz, and *η*_2_Ω_2_ = 2*π* × 4.849 GHz for (**a**–**c**) and Ω_2_ = 2*π* × 7.516 GHz, and *η*_2_Ω_2_ = 2*π* × 5.392 GHz for (**d**–**f**). The initial state is prepared on the state $|\tilde{\psi }\mathrm{(0)}\rangle ={|0\rangle }_{r}\otimes |g\rangle$. The other parameters are listed in Table 1.

**Figure 3:**
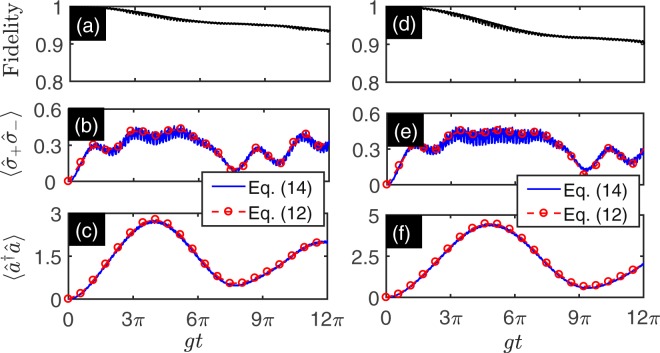
The fidelity and dynamics of simulated QRM with effective coupling ratio $|\tilde{g}/\tilde{\omega }|=1$ (**a**–**c**) and $|\tilde{g}/\tilde{\omega }|=1.2$ (**d**–**f**) as functions of evolution time. (**a**,**d**) Show the fidelity as a function of evolution time governed by the master equation in Eq. (14) and the simulated Hamiltonian given in Eq. (12). (**b**,**e**) Show the qubit excitation number $\langle {\hat{\sigma }}_{+}{\hat{\sigma }}_{-}\rangle$ as a function of evolution time. (**c**,**f**) Show the excitation number of the resonator $\langle {a}^{\dagger }a\rangle$ as function of evolution time. The dynamics is governed by the master equation in Eq. (14) (blue solid line) and the simulated Hamiltonian given in Eq. (12) (red dashed line with circles). The red sideband modulation parameters are chosen as Ω_1_ = 2*π* × 3.2 GHz, and *η*_1_Ω_1_ = 2*π* × 2.296 GHz. The blue sideband modulation parameters are chosen as follows: Ω_2_ = 2*π* × 7.558 GHz, and *η*_2_Ω_2_ = 2*π* × 5.422 GHz for (**a**–**c**) and Ω_2_ = 2*π* × 7.565 GHz, and *η*_2_Ω_2_ = 2*π* × 5.427 GHz for (**d**–**f**). The initial state is prepared on the state $|\tilde{\psi }\mathrm{(0)}\rangle ={|0\rangle }_{r}\otimes |g\rangle$. The other parameters are listed in Table 1.

In Figs 2 and 3, we show the fidelity and dynamics under following four sets parameters: 2(a–c) Ω_2_ = 2*π* × 6.759 GHz, and *η*_2_Ω_2_ = 2*π* × 4.849 GHz; 2(d–f) Ω_2_ = 2*π* × 7.516 GHz, and *η*_2_Ω_2_ = 2*π* × 5.392 GHz; 3(a–c) Ω_2_ = 2*π* × 7.558 GHz, and *η*_2_Ω_2_ = 2*π* × 5.422 GHz; 3(d–f) Ω_2_ = 2*π* × 7.565 GHz, and *η*_2_Ω_2_ = 2*π* × 5.427 GHz. The red sideband modulation parameters are chosen as Ω_1_ = 2*π* × 3.2 GHz, and *η*_1_Ω_1_ = 2*π* × 2.296 GHz. These sets parameters imply the normalized modulation amplitudes *η* = 0.7173. One also can lead to resonant red sideband (i.e., *δ*_1_ = 0) and the detuned blue sideband, and the corresponding detunings read *δ*_2_ = 2*π* × 840.7 MHz, 2*π* × 84.07 MHz, 2*π* × 42.03 MHz and 2*π* × 35.03 MHz. These sets parameters correspond to the four relative coupling strengths $|\tilde{g}/\tilde{\omega }|=0.05$ (Fig. 2(a–c)), $|\tilde{g}/\tilde{\omega }|=0.5$ (Fig. 2(d–f)), $|\tilde{g}/\tilde{\omega }|=1$ (Fig. 3(a–c)) and $|\tilde{g}/\tilde{\omega }|=1.2$ (Fig. 3(d–f)). In the numerical simulation, we take $|\tilde{\psi }\mathrm{(0)}\rangle ={|0\rangle }_{r}\otimes |g\rangle$ as initial state. Figure 2(a,d) show the fidelity as a function of evolution time governed by the master equation in Eq. (14) and the simulated Hamiltonian given in Eq. (12). Figure 2(b,e) show the qubit excitation number $\langle {\hat{\sigma }}_{+}{\hat{\sigma }}_{-}\rangle$ as a function of evolution time. Figure 2(c,f) show the excitation number of the resonator $\langle {\hat{a}}^{\dagger }\hat{a}\rangle$ as a function of evolution time. The dynamics is governed by the master equation in Eq. (14) (blue solid line) and the simulated Hamiltonian given in Eq. (12) (red dashed line with circles). In the case of $|\tilde{g}/\tilde{\omega }|=0.05$, the RWA is valid and the dynamics of qubit and the resonator are dominated by RT. The effects of CRT are very weak, and we can apply RWA safely. In the case of $|\tilde{g}/\tilde{\omega }|=0.5$, the RWA is not valid and the effects of CRT cannot be ignored. The qubit and resonator can be excited simultaneously. The Fig. 3 shows the fidelity and dynamics when $|\tilde{g}/\tilde{\omega }|=1$ (Fig. 3(a–c)) and $|\tilde{g}/\tilde{\omega }|=1.2$ (Fig. 3(d–(f)). In these cases, the relative effective coupling strength reaches 1 and even exceeds 1. The CRT plays an important role in USC and DSC regimes. The exist of CRT makes the total excitation number operator $\hat{N}={\hat{a}}^{\dagger }\hat{a}+{\hat{\sigma }}_{+}{\hat{\sigma }}_{-}$ not a conserved quantity. The excitations of qubit and resonator can be excited from the vacuum. The Fig. 3(d–f) show the fidelity and dynamic when $|\tilde{g}/\tilde{\omega }|=1.2$. In this case, DSC regime is reached.

### The simulation of JC model and anti-JC model

In this subsection, we will show how to obtain the JC model and anti-JC model by tuning the driving parameters to suppress the CRT or RT, respectively. To obtain the JC model, we chosen the modulation parameters as follows: Ω_1_ = 2*π* × 3.2 GHz, *η*_1_Ω_1_ = 2*π* × 3.848 GHz, Ω_2_ = 2*π* × 7.565 GHz, and *η*_2_Ω_2_ = 2*π* × 5.427 GHz, *φ*_1_ = *φ*_2_ = 0. The other parameters are listed in Table 1. These modulation parameters imply *η*_1_ = 1.2024, *η*_2_ = 0.7173, *δ*_1_ = 0, *δ*_2_ = 2*π* × 35.03 MHz and *θ* = 0. One can check that ${\tilde{g}}_{cr}=0$ and the relative coupling strength $|{\tilde{g}}_{r}/\tilde{\omega }|=1.137$. In this case, the rotating term is suppressed to zero and the effective Hamiltonian reduced to the following the JC model in DSC regime

16 $${H}_{{\rm{JC}}}=\tilde{\omega }{a}^{\dagger }a+\frac{\tilde{\varepsilon }}{2}{\hat{\sigma }}_{z}+{\tilde{g}}_{r}(a{\hat{\sigma }}_{+}+{a}^{\dagger }{\hat{\sigma }}_{-}).$$

**Table 1:** The system parameters are listed.

*ε*/2*π*	*ω*/2*π*	*g*/2*π*	*γ*/2*π*	*κ*/2*π*
5.4 GHz	2.2 GHz	70 MHz	12 KHz	50 KHz

**Figure 4:**
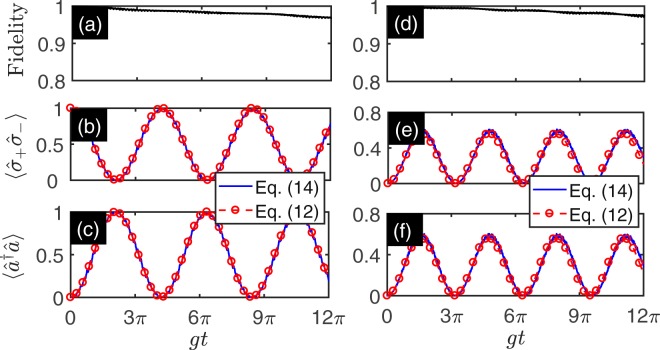
The fidelity and dynamics of simulated QRM with effective coupling ratio $|{\tilde{g}}_{r}/\tilde{\omega }|=1.137$ (**a**–**c**) and $|{\tilde{g}}_{cr}/\tilde{\omega }|=1.137$ (**d**–**f**) as functions of evolution time. (**a**,**d**) Show the fidelity as a function of evolution time are governed by the master equation in Eq. (14) and the simulated Hamiltonian given in Eq. (12). (**b**,**e**) Show the qubit excitation number $\langle {\hat{\sigma }}_{+}{\hat{\sigma }}_{-}\rangle$ as a function of evolution time. (**c**,**f**) Show the excitation number of the resonator $\langle {\hat{a}}^{\dagger }\hat{a}\rangle$ as a function of evolution time. The dynamics is governed by the master equation in Eq. (14) (blue solid line) and the simulated Hamiltonian is given in Eq. (12) (red dashed line with circles). The parameters are taken as follows: Ω_1_ = 2*π* × 3.2 GHz, *η*_1_Ω_1_ = 2*π* × 3.848 GHz, Ω_2_ = 2*π* × 7.565 GHz, and *η*_2_Ω_2_ = 2*π* × 5.427 GHz, *φ*_1_ = *φ*_2_ = 0 for (**a**–**c**) and Ω_1_ = 2*π* × 3.2 GHz, *η*_1_Ω_1_ = 2*π* × 2.296 Ghz, Ω_2_ = 2*π* × 7.565 GHz, and *η*_2_Ω_2_ = 2*π* × 9.096 GHz, *φ*_1_ = *φ*_2_ = 0 for (**d**–**f**). The other parameters are listed in Table 1.

Taking the initial state $|\tilde{\psi }\mathrm{(0)}\rangle ={|0\rangle }_{r}\otimes |e\rangle$, we obtain the fidelity and dynamics of the evolution state governed by the master equation in Eq. (14) and the simulated Hamiltonian given in Eq. (16), which are shown in Fig. 4(a–c). The results show that the numerical simulation agrees well with the exact dynamics. It also shows that there exists the Rabi oscillation between states ${|0\rangle }_{r}\otimes |e\rangle$ and ${|1\rangle }_{r}\otimes |g\rangle$ with period $\pi /|{\tilde{g}}_{r}|$. For the case ${\delta }_{1}\ne 0$ and the initial state $|\tilde{\psi }\mathrm{(0)}\rangle ={|0\rangle }_{r}\otimes |e\rangle$, the period of the Rabi oscillation is $2\pi /\sqrt{4{\tilde{g}}_{r}^{2}+{\delta }_{1}^{2}}$.

To obtain the anti-JC model, we set Ω_1_ = 2*π* × 3.2 GHz, *η*_1_Ω_1_ = 2*π* × 2.296 GHz, Ω_2_ = 2*π* × 7.565 GHz, and *η*_2_Ω_2_ = 2*π* × 9.096 GHz, *φ*_1_ = *φ*_2_ = 0. The other parameters are listed in Table 1. These modulation parameters imply *η*_1_ = 0.7173, *η*_2_ = 1.2024, *δ*_1_ = 0, *δ*_2_ = 2*π* × 35.03 MHz and *θ* = 0. In this case, we can check that ${\tilde{g}}_{r}=0$ and the relative coupling strength $|{\tilde{g}}_{cr}/\tilde{\omega }|=1.137$. The effective Hamiltonian is reduced to the following anti-JC model in DSC regime

17 $${H}_{{\rm{AJC}}}=\tilde{\omega }{\hat{a}}^{\dagger }\hat{a}+\frac{\tilde{\varepsilon }}{2}{\hat{\sigma }}_{z}+{\tilde{g}}_{cr}(\hat{a}{\hat{\sigma }}_{-}+{\hat{a}}^{\dagger }{\hat{\sigma }}_{+}).$$

In the anti-JC model, the rotating term is suppressed to zero and only the CRT remains. We can check the validity and dynamics of the effective Hamiltonian. Let $|\tilde{\psi }\mathrm{(0)}\rangle ={|0\rangle }_{r}\otimes |g\rangle$ be the initial state. The fidelity and dynamics of the evolution state governed by the master equation in Eq. (14) and the simulated Hamiltonian given in Eq. (17) are shown in Fig. 4(d–f). The Fig. 4(d) shows that the numerical simulation agrees well with the exact dynamics. The Fig. 4(e,f) show that excitation number of the resonator and qubit possesses the same behavior. It also shows that there exists the Rabi oscillation between states ${|0\rangle }_{r}\otimes |g\rangle$ and ${|1\rangle }_{r}\otimes |e\rangle$ with period $2\pi /\sqrt{4{\tilde{g}}_{cr}^{2}+{\delta }_{2}^{2}}$. For the case *δ*_2_ = 0, the period of the Rabi oscillation is $\pi /|{\tilde{g}}_{cr}|$. Such behavior is induced by pure effect of CRT and have been studied in ref.^90^.

### The simulation of degenerate AQRM

In this subsection, we will simulate the dynamics of the AQRM. For simplify, we choose the modulation parameters are as follows: Ω_1_ = 2*π* × 3.2 GHz, *η*_1_Ω_1_ = 2*π* × 2.296 GHz, Ω_2_ = 2*π* × 7.6 GHz, *φ*_1_ = *φ*_2_ = 0, and the blue sideband modulation amplitude ranges from 0 to 2*π* × 9.138 GHz. The other parameters are given in Table 1. Then we can obtain *δ*_1_ = *δ*_2_ = 0, *η*_1_ = 0.7173 and *θ* = 0. The normalized amplitude of blue sideband ranges from 0 to 1.2024. In this case, only interaction terms remain and the effective Hamiltonian reduces to the following degenerate AQRM

18 $${\hat{H}}_{{\rm{DAQRM}}}={\tilde{g}}_{r}(\hat{a}{\hat{\sigma }}_{+}+{\hat{a}}^{\dagger }{\hat{\sigma }}_{-})+{\tilde{g}}_{cr}(\hat{a}{\hat{\sigma }}_{-}+{\hat{a}}^{\dagger }{\hat{\sigma }}_{+}).$$

**Figure 5:**
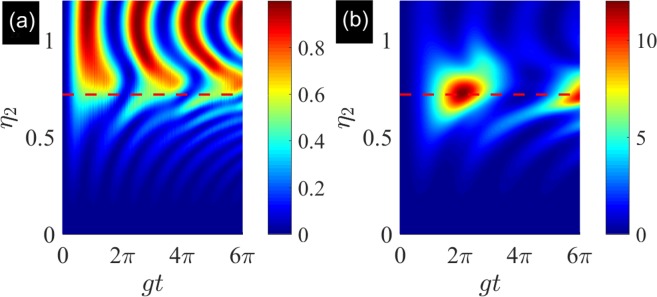
The dynamics of simulated degenerate AQRM as a function of evolution time and *η*_2_. (**a**) Shows the excitation of qubit $\langle {\hat{\sigma }}_{+}{\hat{\sigma }}_{-}\rangle$ as a function of evolution time and *η*_2_. (**b**) Shows the excitation of resonator $\langle {\hat{a}}^{\dagger }\hat{a}\rangle$ as a function of evolution time and *η*_2_.The parameters are taken as follows: Ω_1_ = 2*π* × 3.2 GHz, *η*_1_Ω_1_ = 2*π* × 2.296 GHz, Ω_2_ = 2*π* × 7.6 GHz, *φ*_1_ = *φ*_2_ = 0, and the blue sideband modulation amplitude ranges from 0 to 2*π* × 9.138 GHz (i.e., *η*_2_ ranges from 0 to 1.2024). The other parameters are given in Table 1. The initial state is chosen as $|\tilde{\psi }\mathrm{(0)}\rangle ={|0\rangle }_{r}\otimes |g\rangle$. The red dashed line is plotted for *η*_2_ = 0.7173. The evolution states are governed by the master Eq. (14).

We check that the simulated Hamiltonian varies from JC model to anti-JC model by tuning the normalized amplitude *η*_2_. Let $|\tilde{\psi }\mathrm{(0)}\rangle ={|0\rangle }_{r}\otimes |g\rangle$ be the initial state. We can obtain the dynamics of the evolution states governed by the master equation in Eq. (13). The excitations of qubit and resonator as a function of evolution time and *η*_2_ are shown in Fig. 5. The Fig. 5(a) shows the excitation of qubit $\langle {\hat{\sigma }}_{+}{\hat{\sigma }}_{-}\rangle$ as a function of evolution time and *η*_2_. When ${\eta }_{2}\ll 1$, we can check that ${\tilde{g}}_{cr}$ approaches to zero and rotating term dominates the dynamics. The qubit and resonator are not excited in the evolution process. If we increase the normalized amplitude *η*_2_, the effects of the CRT emerge. In this regime, the qubit and resonator are excited in the evolution process. When *η*_2_ = 0.7173 (red dashed line), the ration of the RT and CRT approaches to 1. In this regime, the RT and CRT dominate the dynamics of the evolution. The Fig. 5(b) shows the excitation number of resonator $\langle {\hat{a}}^{\dagger }\hat{a}\rangle$. When *η*_2_ = 0.7173 (red dashed line), the excitation number reaches its maximum value in the evolution process, which originates from the competition of RT and CRT. When *η*_2_ reaches 1.2024, we can check that when ${\tilde{g}}_{r}$ approaches to zero, the CRT dominates the evolution. The higher excitation number of the resonator can be excited. The dynamics of the qubit and resonator show the periodic oscillation behavior. The results show that we can drive the system from JC regime to anti-JC regime through quantum Rabi regime (indicated by red dashed line).

## Some Applications on the Quantum Information Theory

Our scheme could be utilized as a candidate platform to implement the quantum information and computation device. As an example, we show the generations of Schrödinger cat states and quantum gate. For this purpose, we first generalize our scheme to the multi-qubit case^12^. Considering *N* qubits coupled to a resonator, we can obtain the simulated anisotropic quantum Dicke model with the same treatment. We assume all the qubits possess the same energy split (*i.e*., *ε*_*i*_ = *ε*) and the periodic driving fields described in Eq. (2c) act on all the qubits. By means of the same approach, we can obtain the simulated anisotropic quantum Dicke model. The simulated anisotropic quantum Dicke model in the interaction picture reads

19 $${\hat{H}}_{{\rm{DM}}}={\tilde{g}}_{r}\hat{a}{\hat{J}}_{+}\,{e}^{-i({\delta }_{1}t+{\varphi }_{1})}+{\tilde{g}}_{cr}\hat{a}{\hat{J}}_{-}\,{e}^{-i({\delta }_{2}t+{\varphi }_{2})}+{\rm{H}}.\,{\rm{c}}.\,,$$

where ${\hat{J}}_{\pm }={\sum }_{i}^{N}\,{\hat{\sigma }}_{i\pm }$ and ${\hat{J}}_{z}=\frac{1}{2}[{\hat{J}}_{+},{\hat{J}}_{-}]$. If we set the detunings of the blue and red sidebands *δ*_1_ = *δ*_2_ = *δ*, we obtain the degenerate two-level system (*i.e*., $\tilde{\varepsilon }=0$), and the effective frequency of resonator is $\tilde{\omega }=\delta$. We also can adjust the normalized amplitudes of the driving fields to make ${\tilde{g}}_{r}={\tilde{g}}_{cr}=\tilde{g}$. For simplicity, we set amplitudes *η*_1_ = *η*_2_ and initial driving phases *φ*_*i*_ = 0. In this case, the simulated Hamiltonian in the interaction picture reduces to the following form

20 $${\hat{H}}_{{\rm{DM}}}=\tilde{g}({\hat{a}}^{\dagger }{e}^{i\tilde{\omega }t}+\hat{a}{e}^{-i\tilde{\omega }t}){\hat{J}}_{x}.$$

The evolution operator for the Hamiltonian in Eq. (20), which could be obtained by means of the Magnus expansion, reads^91^

21 $${\mathscr{U}}(t)=\exp \,[i\phi (t){\hat{J}}_{x}^{2}]D[\xi (t){\hat{J}}_{x}],$$

where $D(\xi )=\exp \,(\xi {\hat{a}}^{\dagger }-{\xi }^{\ast }\hat{a})$, $\xi (t)=(\tilde{g}/\tilde{\omega })(1-{e}^{i\tilde{\omega }t})$ and $\phi (t)={(\tilde{g}/\tilde{\omega })}^{2}(\tilde{\omega }t-\,\sin \,(\tilde{\omega }t))$. Based on the dynamics of this effective Hamiltonian, the Schrödinger cat states and quantum gate can be generated.

### The generation of Schrödinger cat states

Superposition of coherent states plays an important role in quantum theory^79,80,92–94^. In this subsection, we consider how to generate superposition of coherent states for a single-qubit case. Assuming the initial state prepared on $|\tilde{\psi }\mathrm{(0)}\rangle ={|0\rangle }_{r}\otimes |g\rangle$, we obtain the evolution state as follows

22 $$|\tilde{\psi }(t)\rangle =\frac{{e}^{i\phi (t)}}{\sqrt{2}}({|\xi (t)\rangle }_{r}\otimes |+\rangle -{|-\xi (t)\rangle }_{r}\otimes |-\rangle ),$$

where $|\,\pm \,\rangle =\frac{1}{\sqrt{2}}(|e\rangle \pm |g\rangle )$ are the eigenstates of ${\hat{\sigma }}_{x}$ and ${|\pm \xi (t)\rangle }_{r}=D[\xi (t)]{|0\rangle }_{r}$ are the coherent states with amplitude ±*ξ*(*t*). In the basis $|e\rangle$ and $|g\rangle$, the above state can be rewritten as following form

23 $$|\tilde{\psi }(t)\rangle =\frac{{e}^{i\phi (t)}}{2}({|{{\mathscr{C}}}_{-}(t)\rangle }_{r}\otimes |e\rangle +{|{{\mathscr{C}}}_{+}(t)\rangle }_{r}\otimes |g\rangle ),$$

where $|{{\mathscr{C}}}_{\pm }(t)\rangle ={{\mathscr{N}}}_{\pm }(|\xi (t)\rangle \pm |-\xi (t)\rangle )$ with normalization coefficients ${{\mathscr{N}}}_{\pm }=\sqrt{2(1\pm \exp \,(\,-\,\mathrm{2|}\xi (t){|}^{2}))}$. Performing a projection measurement on the states $|e\rangle$ and $|g\rangle$, we obtain the states $|{{\mathscr{C}}}_{+}(t)\rangle$ and $|{{\mathscr{C}}}_{-}(t)\rangle$, which correspond to the even and odd Schrödinger cat states. The magnitude of the displacement for $|{{\mathscr{C}}}_{\pm }(t)\rangle$ is $|\xi (t)|=\mathrm{2|}(\tilde{g}/\tilde{\omega })\,\sin \,(\tilde{\omega }t\mathrm{/2})|$. When the evolution time ${t}_{0}=\pi /\tilde{\omega }$, the magnitude of the displacement reaches its maximum value $\mathrm{2|}\tilde{g}/\tilde{\omega }|$.

### The implementation of quantum gate

In this subsection, we consider two-qubit case. Assuming the evolution time $T=2\pi /\tilde{\omega }$, we obtain *ξ*(*T*) = 0 and $\phi (T)=2\pi {(\tilde{g}/\tilde{\omega })}^{2}$. The evolution operator is reduced to the following form

24 $${\mathscr{U}}(T)=\exp \,[i\phi (T){J}_{x}^{2}].$$

where ${J}_{x}={\hat{\sigma }}_{1x}+{\hat{\sigma }}_{2x}$ for two-qubit case. The Eq. (24) can be rewritten as the form ${\mathscr{U}}(T)=\,\cos \,{\vartheta } {\mathcal I} +i\,\sin \,\vartheta {\hat{\sigma }}_{1x}{\hat{\sigma }}_{2x}$, where $\vartheta =2\phi (T)$ and ${\mathcal I}$ is identity operator for two-qubit. Here, we have omitted the total phase factor. To assess the capacity of the quantum gate, Zanardi *et al*. introduced the entangling power^95,96^. The entangling power for this unitary operator reads ${e}_{p}({\mathscr{U}})=\frac{2}{9}{\sin }^{2}\,\mathrm{(2}\vartheta )$. So the evolution operator can be viewed as a nontrival two-qubit quantum gate when $\theta \ne \frac{k}{2}\pi$ (*k* is integer). When $\vartheta =\pi /4$ (*i.e*., $\tilde{g}/\tilde{\omega }=0.25$), the quantum gate reads ${\mathscr{U}}(T)=\frac{1}{\sqrt{2}}( {\mathcal I} +i{\hat{\sigma }}_{1x}{\hat{\sigma }}_{2x})$. Such quantum gate is local equivalent to the control-not (CNOT) gate^97,98^. The equivalent relation reads

25 $${\rm{CNOT}}=({u}_{1}\otimes {u}_{2}){\mathscr{U}}(T)({u}_{3}\otimes {u}_{4}),$$

where local unitary operators are as follows

26 $${u}_{1}=\frac{1}{\sqrt{2}}(\begin{array}{cc}-1 & 1\\ 1 & 1\end{array}),\,{u}_{2}=(\begin{array}{cc}1 & 0\\ 0 & 1\end{array}),\,{u}_{3}=\frac{1}{\sqrt{2}}(\begin{array}{cc}-1 & -i\\ 1 & -i\end{array}),\,{u}_{4}=\frac{1}{\sqrt{2}}(\begin{array}{cc}1 & i\\ i & 1\end{array}).$$

## Discussion

In conclusion, we have proposed a method to simulate a tunable AQRM, which is achieved by driving the qubit(s) with two-tone periodic driving fields. We have analyzed the parameter conditions under which this proposal works well. By choosing proper modulation frequencies and amplitudes, the coupling constants of RT or CRT can be suppressed to zero, respectively. Consequently, we study the dynamics induced by CRT or RT correspondingly. In addition, we have also discussed the applications of our scheme to the generations of quantum gate and Schrödinger cat states. This proposal provides us with a reliable approach for studying the effects of RT and CRT in different regimes individually. Although we explore the scheme with the circuit QED system, which could be implemented in other systems, e.g., cavity QED and trapped ion systems. The presented proposal will pave a way to further study the stronger light-matter interaction in a system whose coupling strength is far away from the USC and DSC regimes in quantum optics.

Extensions of presented scheme to a variety of physically relevant systems, such as multi-qubit and multi-mode fields interaction system and the system coupling with the environments, deserve future investigations.

## References

[1] Rabi II (1936). On the Process of Space Quantization. Phys. Rev..

[2] Rabi II (1937). Space Quantization in a Gyrating Magnetic Field. Phys. Rev..

[3] Braak D (2011). Integrability of the Rabi model. Phys. Rev. Lett..

[4] Jaynes ET, Cummings FW (1963). Comparison of quantum and semiclassical radiation theories with application to the beam maser. Proc. IEEE.

[5] Shore BW, Knight PL (1993). The Jaynes-Cummings model. J. Mod. Opt..

[6] Meekhof DM, Monroe C, King BE, Itano WM, Wineland DJ (1996). Generation of Nonclassical Motional States of a Trapped Atom. Phys. Rev. Lett..

[7] Leibfried D, Blatt R, Monroe C, Wineland DJ (2003). Quantum Dynamics of Single Trapped Ions. Rev. Mod. Phys..

[8] Häffner HH, Roos CF, Blatt R (2008). Quantum Computing with Trapped Ions. Phys. Rep..

[9] Lv D (2017). Reconstruction of the Jaynes-Cummings Field State of Ionic Motion in a Harmonic Trap. Phys. Rev. A.

[10] Blais A, Huang R-S, Wallraff A, Girvin SM, Schoelkopf RJ (2004). Cavity quantum electrodynamics for superconducting electrical circuits: An architecture for quantum computation. Phys. Rev. A.

[11] Wallraff A (2004). Strong coupling of a single photon to a superconducting qubit using circuit quantum electrodynamics. Nature.

[12] Blais A (2007). Quantum information processing with circuit quantum electrodynamics. Phys. Rev. A.

[13] Miller R (2005). Trapped Atoms in Cavity QED: Coupling Quantized Light and Matter. J. Phys. B.

[14] Walther H, Varcoe BTH, Englert BG, Becker T (2006). Cavity Quantum Electrodynamics. Rep. Prog. Phys..

[15] Todorov Y (2010). Ultrastrong Light-Matter Coupling Regime with Polariton Dots. Phys. Rev. Lett..

[16] Deng CQ, Orgiazzi J-L, Shen FR, Ashhab S, Lupascu A (2015). Observation of Floquet States in a Strongly Driven Artificial Atom. Phys. Rev. Lett..

[17] Forn-Díaz P (2010). Observation of the Bloch-Siegert Shift in a Qubit-Oscillator System in the Ultrastrong Coupling Regime. Phys. Rev. Lett..

[18] Niemczyk T (2010). Circuit quantum electrodynamics in the ultrastrong-coupling regime. Nat. Phys..

[19] Scalari G (2012). Ultrastrong Coupling of the Cyclotron Transition of a 2D Electron Gas to a THz Metamaterial. Science.

[20] Anappara AA (2009). Signatures of the ultrastrong light-matter coupling regime. Phys. Rev. B.

[21] Günter G (2009). Sub-cycle switch-on of ultrastrong light-matter interaction. Nature.

[22] Fedorov A (2010). Strong Coupling of a Quantum Oscillator to a Flux Qubit at Its Symmetry Point. Phys. Rev. Lett..

[23] Muravev VM, Andreev IV, Kukushkin IV, Schmult S, Dietsche W (2011). Observation of hybrid plasmon-photon modes in microwave transmission of coplanar microresonators. Phys. Rev. B.

[24] Schwartz T, Hutchison JA, Genet C, Ebbesen TW (2011). Reversible Switching of Ultrastrong Light-Molecule Coupling. Phys. Rev. Lett..

[25] Geiser M (2012). Ultrastrong Coupling Regime and Plasmon Polaritons in Parabolic Semiconductor Quantum Wells. Phys. Rev. Lett..

[26] Goryachev M (2014). High-Cooperativity Cavity QED with Magnons at Microwave Frequencies. Phys. Rev. Applied.

[27] Zhang Q (2016). Collective, Coherent, and Ultrastrong Coupling of 2D Electrons with Terahertz Cavity Photons. Nature Physics.

[28] Chen Z (2017). Single-photon-driven high-order sideband transitions in an ultrastrongly coupled circuit-quantum-electrodynamics system. Phys. Rev. A.

[29] Solano E, Agarwal GS, Walther H (2003). Strong-Driving-Assisted Multipartite Entanglement in Cavity QED. Phys. Rev. Lett..

[30] Yoshihara F (2017). Superconducting qubit-oscillator circuit beyond the ultrastrong-coupling regime. Nature Phys..

[31] Solano E (2011). The dialogue between quantum light and matter. Physics.

[32] Garziano L (2016). One photon can simultaneously excite two or more atoms. Phys. Rev. Lett..

[33] Wang X, Miranowicz A, Li H-R, Nori F (2017). Observing pure effects of counter-rotating terms without ultrastrong coupling: A single photon can simultaneously excite two qubits. Phys. Rev. A.

[34] Ridolfo A, Leib M, Savasta S, Hartmann MJ (2012). Photon Blockade in the Ultrastrong Coupling Regime. Phys. Rev. Lett..

[35] Ridolfo A, Savasta S, Hartmann MJ (2013). Nonclassical Radiation from Thermal Cavities in the Ultrastrong Coupling Regime. Phys. Rev. Lett..

[36] Law CK (2013). Vacuum Rabi oscillation induced by virtual photons in the ultrastrong-coupling regime. Phys. Rev. A.

[37] Cao XF, You JQ, Zheng H, Kofman AG, Nori F (2010). Dynamics and quantum Zeno effect for a qubit in either a low- or high-frequency bath beyond the rotating-wave approximation. Phys. Rev. A.

[38] Ai Q, Li Y, Zheng H, Sun CP (2010). Quantum anti-Zeno effect without rotating wave approximation. Phys. Rev. A.

[39] Li PB, Gao SY, Li FL (2012). Engineering two-mode entangled states between two superconducting resonators by dissipation. Phys. Rev. A.

[40] Wang X (2014). Preparing ground states and squeezed states of nanomechanical cantilevers by fast dissipation. Phys. Rev. A.

[41] Reiter F, Tornberg L, Johansson G, Sørensen AS (2013). Steady-state entanglement of two superconducting qubits engineered by dissipation. Phys. Rev. A.

[42] He S, Zhao Y, Chen QH (2014). Absence of collapse in quantum Rabi oscillations. Phys. Rev. A.

[43] Rossatto DZ (2016). Entangling polaritons via dynamical Casimir effect in circuit quantum electrodynamics. Phys. Rev. B.

[44] Felicetti S (2014). Dynamical Casimir Effect Entangles Artificial Atoms. Phys. Rev. Lett..

[45] Kyaw TH, Herrera-Martí DA, Solano E, Romero G, Kwek L-C (2015). Creation of quantum error correcting codes in the ultrastrong coupling regime. Phys. Rev. B.

[46] Romero G, Ballester D, Wang YM, Scarani V, Solano E (2012). Ultrafast Quantum Gates in Circuit QED. Phys. Rev. Lett..

[47] Wang YM, Guo C, Zhang G-Q, Wang GC, Wu CF (2017). Ultrafast quantum computation in ultrastrongly coupled circuit QED systems. Sci. Rep..

[48] Felicetti S (2015). Spectral collapse via two-phonon interactions in trapped ions. Phys. Rev. A.

[49] Lv DS (2018). Quantum simulation of the quantum Rabi model in a trapped ion. Phys. Rev. X.

[50] Pedernales JS (2015). Quantum Rabi Model with Trapped Ions. Sci. Rep..

[51] Cheng X-H (2018). Nonlinear Quantum Rabi Model in Trapped Ions. Phys. Rev. A.

[52] Leroux C, Govia LCG, Clerk AA (2018). Enhancing Cavity Quantum Electrodynamics via Antisqueezing: Synthetic Ultrastrong Coupling. Phys. Rev. Lett..

[53] Braumüller J (2017). Analog quantum simulation of the Rabi model in the ultra-strong coupling regime. Nature Communications.

[54] Li J (2013). Motional Averaging in a Superconducting Qubit. Nature Communications.

[55] Ballester D, Romero G, Garcá-Ripoll JJ, Deppe F, Solano E (2012). Quantum Simulation of the Ultrastrong-Coupling Dynamics in Circuit Quantum Electrodynamics. Phys. Rev. X.

[56] Mezzacapo A (2014). Digital Quantum Rabi and Dicke Models in Superconducting Circuits. Sci. Rep..

[57] Langford NK (2017). Experimentally simulating the dynamics of quantum light and matter at ultrastrong coupling. Nature Communications.

[58] Crespi A, Longhi S, Osellame R (2012). Photonic Realization of the Quantum Rabi Model. Phys. Rev. Lett..

[59] Felicetti S, Romero G, Solano E, Sabín C (2017). Quantum Rabi model in a superfluid Bose-Einstein condensate. Phys. Rev. A.

[60] Tomka M, Araby OE, Pletyukhov M, Gritsev V (2014). Exceptional and regular spectra of a generalized Rabi model. Phys. Rev. A.

[61] Shen LT, Yang ZB, Lu M, Chen RX, Wu HZ (2014). Ground state of the asymmetric Rabi model in the ultrastrong coupling regime. Appl. Phys. B.

[62] Zhang GF, Zhu HJ (2015). Analytical Solution for the Anisotropic Rabi Model: Effects of Counter-Rotating Terms. Sci. Rep..

[63] Zhang YY, Chen XY (2017). Analytical solutions by squeezing to the anisotropic Rabi model in the nonperturbative deep-strong-coupling regime. Phys. Rev. A.

[64] Xie QT, Cui S, Cao JP, Amico L, Fan H (2014). Anisotropic Rabi model. Phys. Rev. X.

[65] Zhang YY (2016). Generalized squeezing rotating-wave approximation to the isotropic and anisotropic Rabi model in the ultrastrong-coupling regime. Phys. Rev. A.

[66] Yu Y-X, Ye J, Liu W-M (2013). Goldstone and Higgs modes of photons inside a cavity. Sci. Rep..

[67] Liu MX (2017). Universal scaling and critical exponents of the anisotropic quantum Rabi model. Phys. Rev. Lett..

[68] Shen LT, Yang ZB, Wu HZ, Zheng SB (2017). Quantum phase transition and quench dynamics in the anisotropic Rabi model. Phys. Rev. A.

[69] Joshi C, Larson J, Spiller TP (2016). Quantum state engineering in hybrid open quantum systems. Phys. Rev. A.

[70] Wang ZH, Zheng Q, Wang XG, Li Y (2016). The energy level crossing behavior and quantum Fisher information in a quantum well with spin-orbit coupling. Sci. Rep..

[71] Schiemann J, Egues JC, Loss D (2003). Variational study of the *v* = 1 quantum Hall ferromagnet in the presence of spin-orbit interaction. Phys. Rev. B.

[72] Baksic A, Ciuti C (2014). Controlling Discrete and Continuous Symmetries in Superradiant Phase Transitions with Circuit QED Systems. Phys. Rev. Lett..

[73] Yang WJ, Wang XB (2017). Ultrastrong-coupling quantum-phase-transition phenomena in a few-qubit circuit QED system. Phys. Rev. A.

[74] Wang YM (2018). Quantum criticality and state engineering in the simulated anisotropic quantum Rabi model. New J. Phys..

[75] Aedo I, Lamata L (2018). Analog quantum simulation of generalized Dicke models in trapped ions. Phys. Rev. A.

[76] Strand JD (2013). First-order sideband transitions with flux-driven asymmetric transmon qubits. Phys. Rev. B.

[77] Navarrete-Benlloch CN, García-Ripoll JJ, Porras D (2014). Inducing Nonclassical Lasing via Periodic Drivings in Circuit Quantum Electrodynamics. Phys. Rev. Lett..

[78] Yan YY, Lü ZG, Zheng H (2015). Bloch-Siegert shift of the Rabi model. Phys. Rev. A.

[79] Liao JQ, Huang JF, Tian L (2016). Generation of macroscopic Schrödinger-cat states in qubit-oscillator systems. Phys. Rev. A.

[80] Huang JF, Liao JQ, Tian L, Kuang LM (2017). Manipulating counter-rotating interactions in the quantum Rabi model via modulation of the transition frequency of the two-level system. Phys. Rev. A.

[81] Silveri MP, Tuorila JA, Thuneberg EV, Paraoanu GS (2017). Quantum systems under frequency modulation. Rep. Prog. Phys..

[82] Basak S, Chougale Y, Nath R (2018). Periodically Driven Array of Single Rydberg Atoms. Phys. Rev. Lett..

[83] Xue ZY, Zhou J, Wang ZD (2015). Universal holonomic quantum gates in decoherence-free subspace on superconducting circuits. Phys. Rev. A.

[84] Chen T, Zhang J, Xue ZY (2018). Nonadiabatic holonomic quantum computation on coupled transmons with ancillaries. Phys. Rev. A.

[85] Chen T, Xue ZY (2018). Nonadiabatic Geometric Quantum Computation with Parametrically Tunable Coupling. Phys. Rev. Applied.

[86] Colton, D. & Kress, R. *Inverse Acoustic and Electromagnetic Scattering Theory (Applied Mathematical Sciences)*. (Springer, New York, 1998).

[87] Korsch HJ, Klumpp A, Witthaut D (2006). On two-dimensional Bessel functions. J. Phys. A: Math. Gen..

[88] Goerz MH, Motzoi F, Whaley KB, Koch CP (2017). Charting the circuit QED design landscape using optimal control theory. npj Quantum Information.

[89] Hofheinz M (2009). Synthesizing arbitrary quantum states in a superconducting resonator. Nature (London).

[90] Garziano L (2015). Multiphoton quantum Rabi oscillations in ultrastrong cavity QED. Phys. Rev. A.

[91] Blanes S, Casas F, Oteo JA, Ros J (2009). The Magnus expansion and some of its applications. Physics Reports.

[92] Liu Y-X, Wei LF, Nori F (2005). Preparation of macroscopic quantum superposition states of a cavity field via coupling to a superconducting charge qubit. Phys. Rev. A.

[93] Liao JQ, Kuang LM (2008). Nanomechanical resonator coupling with a double quantum dot: quantum state engineering. The European Physical Journal B.

[94] Yin Z-Q, Li T, Zhang X, Duan LM (2013). Large quantum superpositions of a levitated nanodiamond through spin-optomechanical coupling. Phys. Rev. A.

[95] Zanardi P, Zalka C, Faoro L (2000). On the entangling power of quantum evolutions. Phys. Rev. A.

[96] Zanardi P (2001). Entanglement of quantum evolutions. Phys. Rev. A.

[97] Makhlin Y (2002). Nonlocal Properties of Two-Qubit Gates and Mixed Statesand the Optimization of Quantum Computations. Quantum Inf. Process..

[98] Zhang J, Vala J, Sastry S, Whaley KB (2003). Geometric theory of nonlocal two-qubit operations. Phys. Rev. A.

[99] Koch J (2007). Charge-insensitive qubit design derived from the Cooper pair box. Phys. Rev. A.

